# Quantifying differential gene connectivity between disease states for objective identification of disease-relevant genes

**DOI:** 10.1186/1752-0509-5-89

**Published:** 2011-05-31

**Authors:** Jen-hwa Chu, Ross Lazarus, Vincent J Carey, Benjamin A Raby

**Affiliations:** 1 Channing Laboratory, Brigham and Women's Hospital, Harvard Medical School, Boston MA 02115, USA; 2 Division of Pulmonary and Critical Care Medicine, Brigham and Women's Hospital, Boston MA 02115, USA; 3 Center for Genomic Medicine, Brigham and Women's Hospital, Boston MA 02115, USA

## Abstract

**Background:**

Network modeling of whole transcriptome expression data enables characterization of complex epistatic (gene-gene) interactions that underlie cellular functions. Though numerous methods have been proposed and successfully implemented to develop these networks, there are no formal methods for comparing differences in network connectivity patterns as a function of phenotypic trait.

**Results:**

Here we describe a novel approach for quantifying the differences in gene-gene connectivity patterns across disease states based on Graphical Gaussian Models (GGMs). We compare the posterior probabilities of connectivity for each gene pair across two disease states, expressed as a posterior odds-ratio (postOR) for each pair, which can be used to identify network components most relevant to disease status. The method can also be generalized to model differential gene connectivity patterns within previously defined gene sets, gene networks and pathways. We demonstrate that the GGM method reliably detects differences in network connectivity patterns in datasets of varying sample size. Applying this method to two independent breast cancer expression data sets, we identified numerous reproducible differences in network connectivity across histological grades of breast cancer, including several published gene sets and pathways. Most notably, our model identified two gene hubs (MMP12 and CXCL13) that each exhibited differential connectivity to more than 30 transcripts in both datasets. Both genes have been previously implicated in breast cancer pathobiology, but themselves are not differentially expressed by histologic grade in either dataset, and would thus have not been identified using traditional differential gene expression testing approaches. In addition, 16 curated gene sets demonstrated significant differential connectivity in both data sets, including the matrix metalloproteinases, PPAR alpha sequence targets, and the PUFA synthesis pathway.

**Conclusions:**

Our results suggest that GGM can be used to formally evaluate differences in global interactome connectivity across disease states, and can serve as a powerful tool for exploring the molecular events that contribute to disease at a systems level.

## Background

Network and pathway models have been frequently used to describe complex interaction patterns of genes and other types of molecules, and there is increasing recognition that such networks will facilitate a more clear understanding of cellular physiology [1]. Developed using global expression [2], proteomic [3,4], or metabolic [5] measures, the models can be used to characterize the patterns of interaction (gene-gene, gene-protein, etc) that underlie cellular states. Such models have been used to define the complex pathobiology of numerous cancer types [6-8], neurological conditions [9], and metabolic disorders [10]. More recently, models constructed through integration of genotype and expression data have been used to identify disease-susceptibility loci that alter network dynamics [11,12].

Though network models are fairly easy to visualize using graphs, direct comparison of two models (for example, transcriptome networks across disease states), and quantitative measurement of the differences between networks, remains challenging. In recent years there have been growing literature of methodology for such comparisons [13], either for a global scale estimation of overall network similarity [14-16], or for measures of local difference in connectivity for nodes or modules in the network [17-19]. Among the many methods used to infer gene networks are Gaussian Graphical models (GGM) [20-23], including the empirical Bayes methods for fitting Gaussian graphical models [24], which performs well in inferring large-*p *small-*n *gene networks. As a probabilistic method, GGM provides posterior probabilities of gene-gene interaction for each edge in the network, a quantifiable measure of interaction that incorporates the uncertainty of the model. We recently [25] applied the method to build an integrative network based on multiple data sources (i.e. SNP genotypes and gene expression data). We now extend this method to integrate clinical phenotypes, such as disease status, in order to facilitate identification of network modules whose connectivity patterns differ by disease status. Our approach enables direct comparison of two co-expression networks and objective identification of network components that consistently exhibit differential connectivity patterns across disease states. For simplicity we will only consider dichotomous phenotypes, though this method could be extended to categorical or continuous traits as well.

## Methods

**Figure 1:**
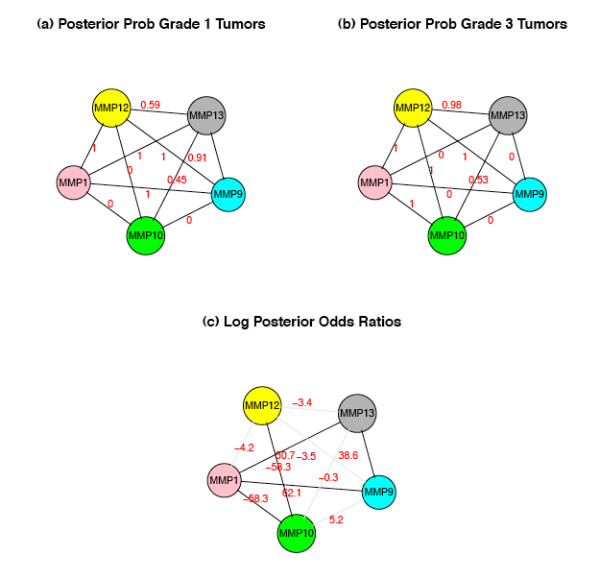
**Determining the posterior odds ratio (postOR)**. Gene network for five genes in the matrix metalloproteinases network determined separately in grade 1 breast cancer samples (a) and grade 3 samples (b). The posterior probabilities of gene-gene connection (in red) determined by GGM, support true edges in both tumor grades between MMP1 and MMP12, and between MMP12 and MMP9 (postProb ~ 1); and a true edge between MMP12 and MMP10 in grade 3 but not grade 1. The log posterior odds ratios of the probabilities (in red in panel c) quantify the magnitude of difference in connectivity across disease states. Data derived from GEO series 2990 [28]. See the results section for detail of the breast cancer data analysis.

**Figure 2:**
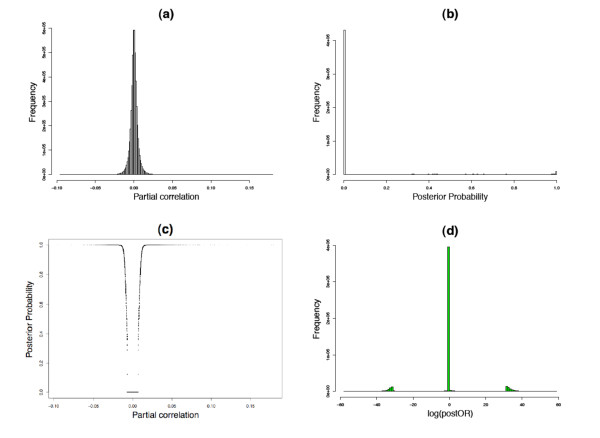
**An example of partial correlations, posterior probabilities and log odds ratios**. (a)Histogram of partial correlation. (b)Histogram of posterior probabilities. (c) The distribution of partial correlations and their corresponding posterior probabilities. (d)Histograms of log odds ratios. Data generated using Illumina HumanRef8 expression data from peripheral blood CD4+ lymphocytes [25].

First we describe the GGM for gene expression data. The expression data matrix *Y *observed here has *G *genes and *N *samples, and the model follows [24] and [25], where *Y *follows a multivariate normal distribution:

where *y_ji _*represents the expression observation for *j*th gene in the *i*th sample, *μ *is the mean vector and Σ is the covariance matrix. The covariance matrix Σ*_Y _*and the partial correlation matrix Π for *Y *are estimated based on the shrinkage estimation described in [26]. The partial correlation Π*_jk _*here represents the conditional dependency between gene *j *and gene *k*, i.e, Π*_jk _*= 0 if the two genes are independent conditional on all other expression values and Π*_jk _*≠ 0 if they are conditionally correlated. Therefore the network estimation problem is reduced to a sequence of *G*(*G *- 1)/2 hypothesis testing problem for Π_*jk *_= 0. Following the mixed model approach in [24] we can calculate the empirical posterior probability that Π*_jk _*≠ 0 for each pair of genes (panel (a) and (b) in Figure 1). Figure 2 shows an example of the distribution of partial correlations and their corresponding posterior probabilities. The partial correlation coefficient Π*_jk _*follows a normal distribution (panel a), but the mixed prior, which assumes that the majority of the gene pairs are not connected, effectively shrinks most of the posterior probabilities toward zero (panel b). We can see in panel (c) as Π*_jk _*grows away from zero the probability of a significant edge quickly approaches 1 and the narrow U-shape demonstrates the ability to identify significant edges for relatively small absolute values of partial correlation coefficients (e.g.~ 0.04-0.05).

Suppose we have the estimation of networks from two different disease groups. If we consider the posterior probability of an edge as a frequency, as if we could actually observe the proportion of samples in the group, then for the two disease groups C and D we can calculate the posterior odds ratio (postOR) for each edge:

where  and  are the posterior probability estimates for the event that an edge exists between gene *j *and gene *k*, in groups C and D, respectively. If  and/or  are zero, we assign them a very small number on the same scale as the smallest non-zero posterior probability to make sure all odds ratios are well-defined. The posterior odds ratios between the disease groups provide a quantitative measure for difference between network connectivity, and the parts of the network where the postORs differ from 1 are likely the parts most relevant to the disease state (panel (c) in Figure 1). Panel (d) in Figure 2 shows a histogram of the log posterior odds ratio, with most of the edges concentrated around zero and relatively few of them way out in the tails, which represent the edges associated with the disease states. The gap from around ±5 to ±30 roughly corresponds to the sharp climb in the posterior probability seen from panel (c) in Figure 2. This pattern has been observed in all data sets that we have analyzed, though the scales in which the extreme observations fall may vary depending on the sample size and the number of genes in the network. As the sample size increases relative to the number of genes, we observe more extreme values of log postORs, in some cases going up to ±50 or 60.

The idea of using posterior odds ratios to quantify differential connectivity can also be generalized to model more focused differential gene connectivity patterns within previously defined sets of genes, including experimentally derived gene networks and canonical pathways. For example, for a given set of genes *A*, we define the differential connectivity score (DC score) as the average absolute differential connectivity, measured by difference in log posterior probability, for all edges comprising set *A*:

which is a good approximation of the average postORs for all edges in the set, as most of the posterior probabilities ,  are close to zero. This gives a reasonable measure of the overall differential connectivity for each gene set.

## Results

### Simulation Study

To assess the theoretical performance of our approach, we performed a series of simulation studies. For each simulation study we first generate two partial correlation matrices representing networks observed in two groups of samples (i.e. "cases" and "controls"), and then generate synthetic expression data sets from them. We then attempt to recover the network using GGM and calculate the postORs for all pairs of genes. To simulate networks most closely resembling real world network data, we set out to develop a set of relatively sparse networks with few strong connections. When generating the partial correlation matrices for the "case" network we therefore follow the same approach in [25], whereby we estimate a connectivity network using an expression dataset generated from peripheral blood CD4+ lymphocytes [25], take the top G genes with the highest correlation, retain correlation coefficients of the top q significant edges and shrink all remaining correlation coefficients to zero. We take G = 100 and q = 77 in our simulation study, which corresponds to about 1.5% of all possible edges (all with posterior probability over 0.95). The "control" networks are from the null model, where the expression data are generated from an independent multivariate distribution and none of the genes are connected. We simulate the expression data with 200 samples in each group and repeat the entire procedure 10 times.

**Figure 3:**
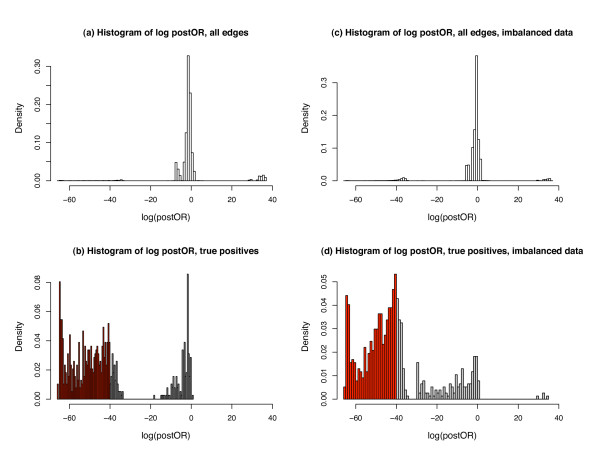
**Histograms of log odds ratios in the simulation studies**. All edges (panel (a) with N = 200 for both data sets and panel (c) for unbalanced samples (N = 200/50)) and true positive edges (i.e. truly differentially connected between disease states, panel (b) for N = 200 and (d) for unbalanced samples). Red shaded results in panel (b) and (d) denote truly differentially connected edges that are not detected at cutoff of logOR = -40 (i.e. false negatives).

**Figure 4:**
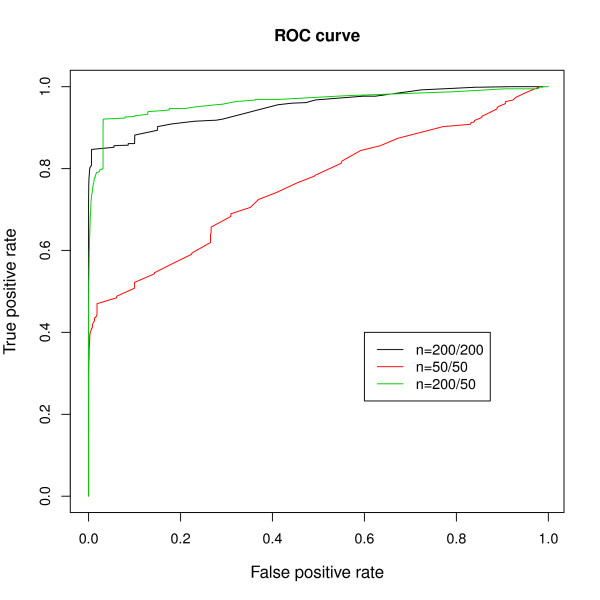
**ROC curves from the simulation studies**. ROC curves from all three cases considered in the simulation studies. Large sample(N = 200/200); small sample (N = 50/50) and unbalanced sample(N = 200/50).

The left panels (a) and (b) in Figure 3 show the histogram of the log posterior odds ratios for all edges (panel a) and for the 1.5% edges that were truly differentiated (panel b). From the right hand side of the panel (a) we see that the log posterior odds ratios from the null edges goes as high as 40. Therefore we take ±40 as the threshold, which gives 72.34% sensitivity and 99.90% specificity for detection of a differentially connected edge. Though we miss a considerable proportion of true edges (shaded in grey in panel b), the very high specificity is particularly encouraging, as it suggests that positive findings are very reliable. Note that even a small reduction in specificity (for example, a 1% increase in the false positive rate) would result in identification of the thousands of spurious differential connections, given the enormous number of pairwise comparisons in any given genome-wide analysis. It is therefore essential to maintain high specificity in this context. We note that for smaller datasets (a simulation with 50 cases and controls), though sensitivity drops considerably (15.06% in our simulation using a cutoff of -40 posterior odds), the high specificity is retained (99.95%). We also considered more realistic scenarios, including situations where both networks (the "cases" and the "controls") contain positive edges and where sample size is uneven between groups, and found very comparable results. For example, right panels (c) and (d) in Figure 3 show an example of unbalanced data, where one set has 200 samples and the other has 50, containing 2.5% and 5% true positive edges, respectively. Using the same threshold of posterior odds at -40 the sensitivity is 40.47% and specificity is 99.65%. Figure 4 shows the ROC curves from all three scenarios considered. We can see that the power varies depending on the sample size and number of variables, but the specificity always stays close to 100%, and the absolute postORs from the null distribution rarely exceed 40. Therefore, we can conclude that in realistic scenarios, though we are not able to identify all truly differentially connected edges, those edges that are declared as differentially connected between states are very likely to be true findings.

**Figure 5:**
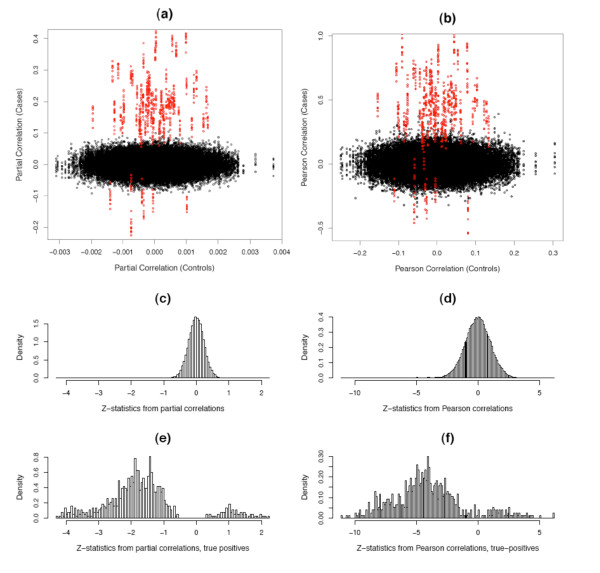
**Partial correlations and Pearson correlations**. Panel (a-b) show the distribution of Fisher-transformed partial correlation (a) and Pearson correlation coefficients (b) from the simulated "case" and "control" data sets. The red dots represents the differentially connected edges. Panel (c-f) show the histograms of z-statistics , where *z*_*ijc *_and *z_ijd _*are the Fisher-transformed correlation coefficients from the "case" and "control" data sets, respectively. Panel (c-d) are for all edges and panel (e-f) are for the truly differentially connected edges only.

Alternatively, we could compare the partial correlations or Pearson correlations between the "cases" and "controls", as shown in Figure 5. In both cases the truly differentially connected edges seem well-separated from the unconnected edges (panel a-b), though from the histogram of the z-statistics (panel c-f) we can see that the true positive edges from partial correlations separate better (have less overlapping with the true negative edges) than the Pearson correlations, which are routinely used to infer gene networks [18,27]. Notice for the correlation coefficients we still need to apply arbitrary thresholds [13], as we do not have repeated measurement for the correlations for each individual edge. Compared to Figure 3 we can see that the postORs from the empirical Bayes method, which takes into consideration the sparsity of real gene network, allow us to effectively separate the truly differentially connected edges from others.

### Breast Cancer Study

We now demonstrate the application of our method to real data sets. The main results will be focused on the comparison between two independent gene expression data sets from breast cancer tissues of varying histological grade available through the Gene Expression Omnibus (GEO series GSE2990 and GSE6532). The GSE2990 series consists of Affymetrix Human Genome U133A Array data for 189 breast tumor samples from the National Cancer Institute database [28], from which we selected 100 estrogen receptor-positive (ER+) samples with histological grades 1 (n = 61) and 3 (n = 39). The GSE6532 series contains several independent validation sets generated using Affymetrix U133PLUS2 GeneChips and described in [29], from which we used the 33 samples from Guy's Hospital, UK (17 grade 1 and 16 grade 3). These data sets were selected based on sample sizes and availability of clinical phenotypes. Using the R package genefilter[30], we applied the non-specific gene ltering [31] on both data sets. The resultant data set consisted of 1,445 RefSeq-annotated genes with interquartile ranges (IQR) in the upper 50% for both data sets.

We applied our method sequentially to define, in each dataset, the differences in network connectivity patterns observed across breast cancers of different histological grades. The two datasets were analyzed separately to enable unbiased evaluation of the reproducibility of findings by our method when applied to biologically independent datasets. We observe a similar pattern to those seen in the simulation studies, with most edges concentrated around zero and relatively few in the extremes. Focusing on the edges with extreme postOR probabilities of differential connectivity between grades (Empirical p-values < 0.001 based on permutation), we found significant overlap across studies. When considering genes exhibiting high degrees of connectivity - so-called hubs [1] defined as genes with at least 30 independent edges - 10 of 33 hubs demonstrating differential connectivity patterns in dataset GSE2990 were also observed in the second dataset GSE6532 (Fisher's exact test, p-value = 1.5 × 10 ^-5^). This high degree of overlap between two independent data sets suggests that the observed differential network connectivity patterns are a reproducible property of complex biological processes such as cancer progression.

We next examined the gene content of the replicated hub genes demonstrating grade-dependent differences in network connectivity, and found that in all but one case (DHRS2), these hub genes have all been previously characterized in expression studies of breast cancer, with many being implicated as critical regulators or markers of metastatic potential and tumor progression (Table 1). That nearly all the identified genes have been previously implicated in breast cancer biology suggests that differential connectivity mapping is exquisitely specific in the identification of biologically relevant genes. We note that the 10th gene, DHRS2, though not previously implicated in studies of breast cancer, has been associated with other estrogen-responsive cancer types of the female reproductive tract, such as endometrial and ovarian cancer [32], suggesting that it too is a true positive finding, and represents a novel breast cancer target.

**Table 1:** Hub list for breast cancer study (GSE2990 and GSE6532) histological grade 1 and 3

Gene	Frequency	Prior evidence for role in breast cancer biology
DHRS2	55/46	Up-regulated in endometrial cancer by the inducer of myometrial infiltration ERM/ETV5 [55]; Protective role against oxidative-stress induced apoptosis in endometrial cancer [55]; Down-regulated in ovarian tumors following cisplatin treatment [32]

CXCL13	30/45	Overexpression in breast cancer tumor tissue, with elevated blood serum levels in patients with metastatic disease [56]

AGTR1	36/42	Overexpressed in subset of estrogen-receptor positive breast cancer; Ectopic overexpression confers a highly invasive phenotype in primary mammary epithelial cells; AGTR1-positive tumor growth reduced by 30% with receptor blockade in xenograft model [54]

KRT15	34/42	Expressed in breast cancer tissue compared to normal breast tissue [57]; Expression associated with increased risk of post-operative breast cancer recurrence [58]

SCGB2A1	38/49	Overexpressed in breast cancer tissue [59]; Associated with mammary gland proliferation and terminal differentiation [60]

MMP12	31/43	Breast tumor transfection of MMP12 reduced endothelial cell invasion and capillary tube formation [50]

PDZK1	47/46	Estrogen-regulated gene expressed in hormone-responsive breast cancer [61]; Correlated with estrogen receptor phenotype [62]; Suppressed with tamoxifen and aromatase inhibitors [63]

BEX1	42/54	BEX2 is overexpressed in a subset of primary breast cancers and mediates nerve growth factor/nuclear factor-kappaB inhibition of apoptosis in breast cancer cell lines. [64]

S100A8	38/42	siRNA-mediated knockdown of S100A8/A9 expression significantly reduced H-Ras-induced invasion/migration; Induction confers the invasive/migratory phenotype [52]; Immunopositivity correlates with mitotic activity, MIB-1 index, HER2 overexpression, node metastasis, and poor prognosis [65]; Associated with transformation and progression of breast cancer cells which is reversed by treatment with silencing inhibitors [53]; Down-regulated in invasive tumors [66]

NAV3	64/42	Differentially expressed in hill-type cancer cells [67]

Frequency denotes the number of differentially connected edges detected in GSE2990 and GSE6532.

**Figure 6:**
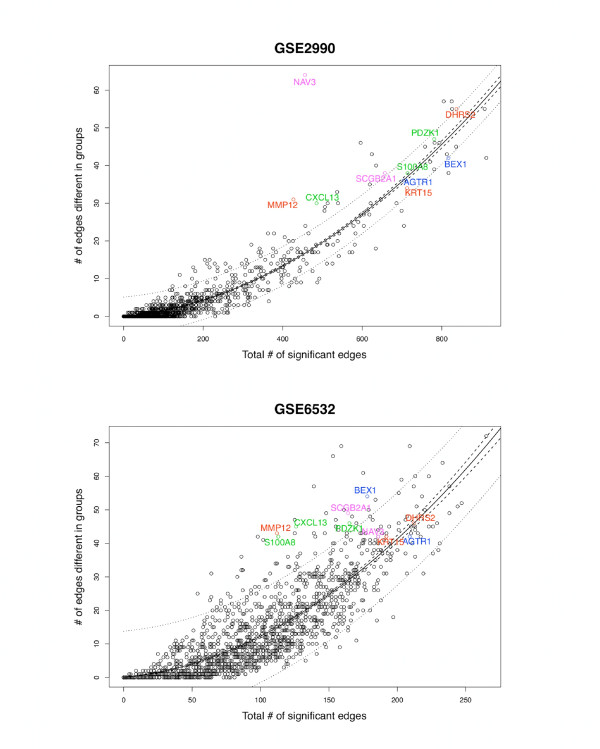
**Differential gene connectivity as a function of overall connectivity for two breast cancer datasets**. The differential gene connectivity for each gene is the number of genes with which the absolute log posterior oddsratio is greater than 55 for GSE2990 and 35 for GSE6532 (thresholds based on ~0.1% percentiles of the null distribution from 100 permutations). The overall connectivity is the number of genes with which the posterior probability of connection is over 90%, which represents about 3.5% of all the edges overall. The solid lines represent the quadratic fitted function and the dashed and dotted lines represent the 95% confidence and prediction intervals, respectively. The 10 genes with 30 or more differential connections in both datasets are labeled. Note that CXCL13 and MMP12 fall outside the 95% confidence and prediction intervals in both datasets.

In contrast to more standard statistical methods, more spurious evidence for differential connectivity might be found, paradoxically, in studies of small sample size when true connections in samples from one disease state are not detected due to low statistical power. We thus performed permutation tests to obtain a null distribution of the number of differential connections for each gene in the two disease states. With 500 permutations, two of the ten genes (CXCL13 and MMP12) were rarely observed in both datasets (0.2% and 0.4%, respectively), and thus can be considered to be reliable hubs demonstrating consistent differential connectivity by histological grade that are not likely observed due to chance. We further note that although there is a strong curvilinear relationship between the total number of significant connections within a network (based on posterior probability thresholds) and the number of differential connections between states (p-values ≈ 0, see Figure 6), we observe that both CXCL13 and MMP12 represent outliers in these distributions of both datasets, exhibiting a higher proportion of differential connections even when accounting for the total number of connections. Therefore, they are unlikely to represent false positive results, and represent high priority targets central to breast cancer grade.

We next examined whether these same genes could be identified using more standard analytic approaches (making our method redundant) or whether our approach provides truly independent information. When we applied traditional differential expression analysis (linear regression as implemented in the R package limma: Linear Models for Microarray Data, [33]) to the datasets, we found that only two of the 10 hub genes - AGTR1 and NAV3 - were themselves differentially expressed by histological grade (FDR adjusted p-value ≤ 0.05). Moreover, none of the 10 differentially connected hub genes were identified as relevant grade-related genes in the original report by [34]. These comparisons suggest that differential connectivity mapping can identify disease relevant genes that would not be found using more traditional approaches. The lack of differential expression for most of the hubs themselves argues that the observed differential connectivity patterns are not primarily due to primary alterations of hub gene expression, but rather due to more subtle changes in expression of numerous genes interacting with these hubs.

**Figure 7:**
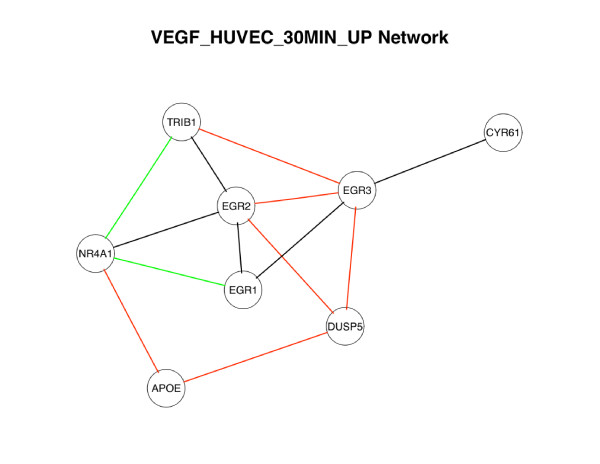
**Differentially connected sub-network of the VEGF_HUVEC_30MIN_UP BroadSet**. VEGF_HUVEC_30MIN_UP is a collection of 24 transcripts significantly upregulated in human umbilical cord endothelial cells at 30 minutes following treatment with VEGF [41]. Network limited to the 8 of 24 transcripts demonstrating differential connectivity in the two breast cancer datasets. Lines denote differentially connected edges observed in GSE2990 (red), GSE6532 (green), or both (black).

We also individually tested each of 5,452 published gene sets comprising the Molecular Signatures Database [[35], MSigDB,] for evidence of differential connectivity in the breast cancer data set. We considered 2,785 MSigDB gene sets that consist of 5 or more genes represented in the breast cancer analysis, and for each gene set we calculated the DC Score. We also performed permutation tests to obtain the null distribution of DC score. DC-scores above the 99% percentile of the null distributions from 100 permutation sets were observed for 108 and 185 Broad Sets in the GSE2990 and GSE6532 breast cancer datasets, respectively, including 80 Broad Sets that exhibited differential network connectivity in both datasets. Additional file 1 (Table S1) details the 16 Broad Sets that reproducibly demonstrated such extreme differential connectivity in both datasets with at least 3 differential connections in each dataset. Most have been implicated in tumor biology, and many of these gene sets have been implicated in breast cancer progression, including chromosomal region 1p33 [36], matrix metalloproteinases (including MMP12), and sequence targets of peroxisome proliferator-activated receptor alpha [37,38]. Potential therapeutic targets were also identified, including subnetworks of the polyunsaturated fatty acid synthesis pathway [39] and of VEGF-induced factors [40] (Figure 7). For example, consistent differential connectivity was noted for a set of genes [[41], Broad Set VEGF_HUVEC_30MIN_UP] upregulated in human umbilical vein endothelial cells (HUVECs) by VEGF, a proangiogenic factors critical to tumor progression and metastasis [40]. The differentially connected sub-network (Figure 7) centers on Cys2-His2 zinc finger transcription factors Early Growth Response 1 and 2 (EGR1 and EGR2). EGR1 and EGR2 directly regulate a series of classical tumor suppressors [42,43], and experimental interference of their expression dramatically alter breast cancer cell growth rates [44,45]. Evidence of differential connectivity was observed for numerous additional gene sets implicated in other carcinomas, though not previously with breast cancer. In response to an anonymous reviewer's suggestion, we also ran an analysis on another breast cancer set, GEO series GSE11121 [46] with Affymetrix Human Genome U133A Array to further confirm the reproducibility of our findings. We selected 29 patients with grade 1 breast cancer and 35 grade 3 breast cancer (ER data unavailable), and compared the networks derived from the two subsets. We found 35 hub genes with over 30 differential connections. Five of them overlap with the hub list from GSE2990(CPB1, PRAME, MMP12, BEX1, NAV3), which use the same platform. Three of them (MMP12, BEX1, NAV3) overlap with both GSE2990 and GSE6532 hub lists. The other gene of interest, CXCL13, also has a large number differential connections (28). These results show strong reproducibility in the third data set, demonstrating that the our findings are not due to platform differences.

## Discussion

The appeal of systems-based or interacteome mapping approaches for the study of disease is steadily increasing with the recognition that non-linear epistatic interaction underlies all but the simplest of biological processes. However, formal identification of biologically relevant interaction patterns imbedded in complex network connectivity maps has been a challenging problem. Several studies have looked at global comparison of the networks based on annotated database, such as GO or KEGG [14-16]. Unlike our method, those previous studies assume complete knowledge of the networks (i.e. they do not accommodate uncertainty in the observed connectivity between nodes). In many instances, however, complete certainty is unattainable. Moreover, these methods are largely global, but do not provide information regarding regional differences (i.e. measures of difference in connectivity between any two nodes in the network). Without a measure of variability of the model, it is not easy to distinguish disease-related genes from those that have neutral roles. There are several methods for comparing region differential connectivity between two networks, based on pair-wise gene co-expression relationships, either at the gene cluster/module level [17,19,47,48] or at the individual gene level [18]. Here we have presented a novel approach that enables direct comparison of two different networks derived from Gaussian graphical model. The key feature of the GGM approach is that the network inference is based on partial correlation (i.e. conditional dependence), which distinguishes direct interactions from indirect ones [24,49]. The postORs from empirical Bayes approach provide an easily interpretable quantitative measure for differential connectivity, allowing search for local differential connectivity either for individual genes, gene pairs, or on a cluster/module level. The method performed well in detecting differential network connectivity in simulations of moderate sample size, compared to other simple methods with Pearson correlations or partial correlations only. In fact, even though the sensitivity was modest, both the simulation studies and the real breast cancer datasets suggest that our approach detects many of the strongest associations with very high specificity.

Application of differential connectivity mapping to the breast cancer data sets provides several important insights, both regarding the utility of this approach to other disease states, and with respect to the importance of network connectivity underlying disease processes such as cancer. With regard to the performance of the method, we first found substantial reproducibility (~30%) in the observed connectivity patterns across the two breast cancer datasets, then similar results were found in the third data set, suggesting network connectivity as a robust, measurable property of complex biological processes. Second, many of the most compelling findings from our analysis (the 10 hubs observed in both datasets) have been previously implicated in breast cancer or other estrogen-responsive cancers, suggesting that the approach is highly specific with regard to biologically relevant findings. Third, as the hubs genes are not always expressed, the majority of the 10 hub genes were not detected using the traditional differential expression approach. Differential connectivity mapping complements differential gene expression analysis and can be used to identify those genes.

Perhaps most importantly, careful review of the specific genes identified suggests that hubs manifesting differential connectivity (or one or more of their connected edges) may represent important candidates for therapeutic targeting. In addition to EGR1 (discussed above), of the 10 hub genes identified, there is experimental evidence for at least three that their targeted manipulation alters the malignant and invasive potential of breast cancer. Matrix metalloprotease 12 (MMP12), a protease that converts plasminogen to angiostatin (a potent inhibitor of angiogenesis), inhibits angiogenesis when overespressed in breast cancer tissue [50]. S100A8, a calcium-binding protein that complexes with S100A9 and whose expression is suppressed by functional BRCA1 [51], is induced by H-Ras to promote malignant potential (tumor cell invasion and migration). Contradictory reports suggest that these malignant properties are either attenuated [52] or enhanced [53] upon siRNA-mediated knockdown of S100A8/A9 expression, suggesting S100A8 as a targetable regulator of malignant potential. Similarly, AGTR1 (one of only two differentially-connected hubs that was also itself differentially expressed across tissue grades) is a potent inducer of invasive phenotypic properties when overexpressed in primary mammary epithelial cells [54]. These effects are inhibited by the AGTR1 antagonist losartan, and FDA-approved medication commonly prescribed for the management of essential hypertension. Consistent with these observations, treatment of xenograft models of breast cancer with losartan reduces tumor growth in AGTR1-positive, but not AGTR1-negative, breast cancers [54]. It is intriguing to speculate whether manipulation of NAV3, the only other gene that displayed both properties of differential connectivity and differential expression across tissue grade, would have similar effects in altering the malignant potential of breast cancers.

## Conclusion

In conclusion, we have developed a highly specific method for the identification of genes that demonstrate differential connectivity across disease states. Though applied here to transcriptome data, this method can be applied more broadly to other types of biological network models, and can serve as a novel approach for the identification of high priority target nodes underlying complex biological processes.

## Authors' contributions

The statistical model and methodology were developed by JC based on the concept by JC and BAR. JC carried out the analysis for simulation and breast cancer data with the support of VJC and RL for statistics and bioinformatics. The manuscript was written by JC and BAR and all co-authors have approved the final version.

## Supplementary Material

Additional file 1 **Broad Sets demonstrating differential connectivity by breast cancer histological grade**. This table includes the 16 Broad Sets that reproducibly demonstrated significant differential connectivity in both GSE2990 and GSE6532 with at least 3 differential connections in each dataset. Click here for file
