# Supplementary material for: Quantifying differential gene connectivity between disease states for objective identification of disease-relevant genes
Source: BMC Syst Biol. 2011 May 31;5:89. doi: 10.1186/1752-0509-5-89 (PMC3128864; doi:10.1186/1752-0509-5-89)
Supplement: Additional file 1 — Broad Sets demonstrating differential connectivity by breast cancer histological grade. This table includes the 16 Broad Sets that reproducibly demonstrated significant differential connectivity in both GSE2990 and GSE6532 with at least 3 differential connections in each dataset. [file 1752-0509-5-89-S1.PDF]

Table S1: Broad Sets demonstrating differential connectivity by breast cancer histological grade

Broad Set (Genes in Set)	Number of genes (Differentially connected / annotated in dataset)		Differential connectivity score (GSE2990/ GSE6532)	Differentially connected edges (GSE2990/ GSE6532)
	GSE2990	GSE6532		
**C1 - Positional Gene Sets**				
Chromosome 1p33	5/12	7/14	0.511/0.360	3/5
Chromosome 6p25	7/13	6/15	0.432/0.274	5/4
Chromosome 4q25	5/12	8/17	0.509/0.240	3/4
**C2 - Curated Gene Sets**				
Matrix metalloproteinases	11/14	5/16	0.371/0.259	17/4
**CP - Canocial Pathways**				
Genes involved in polyunsaturated fatty acid biosynthesis(HSA01040_POLYUNSATURATED_FATTY_ACID_BIOSYNTHESIS)	7/10	7/11	0.750/0.572	5/6
**CGP: chemical and genetic perturbations**				
Downregulated in gastric cancer cell lines reistant to 5-fluorouracil, compared to parent chemosensitive lines(5FU_RESIST_GASTRIC_DN)	6/9	8/15	0.937/0.304	4/6
Downregulated by curcumin at 24 hrs in SW260 colon carcinoma cells(HDACI_COLON_CUR24HRS_DN)	5/11	11/14	0.607/0.336	3/6
Up-regulated in pyloric atrium tissue from Trefoil Factor 2 (Tff2) knockout mice, compared to wild-type controls (TFF2_KO_UP)	7/13	7/15	0.432/0.283	4/6
Up-regulated 30 minutes after VEGF treatment in human umbilical vein endothelial cells (VEGF_HUVEC_30MIN_UP)	8/14	6/15	0.371/0.171	11/7
**C3: motif gene sets**				
**TFT: transcription factor targets**				
Genes with promoter regions [-2kb,2kb] around transcription start site containing the motif CARAACTAGGNCAAAGGTCA which matches annotation for PPARA: peroxisome proliferative activated receptor, alpha (V$PPARA_01)	11/14	8/13	0.371/0.406	11/4
**C4: computational gene sets**				
**CM: Cancer Modules**				
Module 68 (Lung, breast, prostate)	9/12	4/11	0.511/0.564	9/3
Module 78 (Liver)	9/13	6/15	0.432/0.299	7/4
Module 191 (Liver, breast)	7/13	4/14	0.432/0.182	6/4
Module 262 (Liver, breast, leukemia)	7/12	4/14	0.511/0.182	6/4
Module 298 (Liver, lung, breast, prostate)	10/12	8/11	0.511/0.581	11/7
Module 416 (Liver, lung, leukemia)	8/14	6/15	0.371/0.298	6/3
